# Impacts of religious leader training on maternal and child health in underserved communities of Bangladesh

**DOI:** 10.3389/frph.2026.1764081

**Published:** 2026-04-09

**Authors:** Farzana Bari, Farah Naz Rahman, Abu Sayeed Md. Abdullah, Koustuv Dalal, A. K. M. Fazlur Rahman

**Affiliations:** 1 Centre for Injury Prevention and Research, Bangladesh (CIPRB), Dhaka, Bangladesh; 2 School of Public Health and Preventive Medicine, Monash University, Melbourne, VIC, Australia; 3 Maternal and Child Health Division, International Centre for Diarrhoeal Disease Research, Dhaka, Bangladesh; 4 Division of Public Health Science, Department of Health Sciences, Mid Sweden University, Sundsvall, Sweden; 5 University of Johannesburg, Johannesburg, South Africa

**Keywords:** Bangladesh, community health interventions, LMIC, maternal and child health, religious leaders

## Abstract

**Background:**

Religious leaders are trusted in Bangladesh and can promote maternal and child health (MCH)in underserved communities. We assessed the population-level impact of a national training program for religious leaders on knowledge, attitudes, practices, and behaviors (KAPB) related to antenatal care (ANC), facility-delivery, and essential newborn care (ENC).

**Methods:**

We conducted repeated cross-sectional household surveys among imams, female religious-school teachers, and parents of children aged 0–2 years in 17 clusters across eight districts in Bangladesh at baseline (2019, *n* = 3,808) and endline following the training program (2021, *n* = 3,746). Standardized questionnaires captured domain-specific KAPB indicators. Multivariable logistic regression compared endline with baseline, adjusting for age, sex, education, and occupation.

**Results:**

Baseline and endline groups were socio-demographically similar, with small differences in education. Compared with baseline, endline participants showed about two-fold increase in ANC knowledge (aOR 2.3, 95% CI 2.1–2.6), positive attitudes (aOR 1.5, 95% CI 1.4–1.6), and reported ANC use (aOR 2.3, 95% CI 2.1–2.6). Knowledge of home-delivery risks increased markedly (aOR 11.9, 95% CI 9.5–14.9), alongside modest improvements in facility-delivery (aOR 1.5, 95% CI 1.4–1.6). Knowledge of essential newborn care improved greatly (aOR 8.9, 95% CI 7.8–10.1), with better early breastfeeding and bathing practices (aOR 1.8, 95% CI 1.7–2.0). Religious leaders also reported sharing health messages more frequently at endline.

**Conclusion:**

Despite being a quasi-experimental study that limits causal inference, training religious leaders was found to be associated with improved community knowledge and practices across the MCH care continuum. Findings suggests that integrating such engagement into national health communication platforms may enhance coverage in underserved settings. A key limitation of this study is its quasi-experimental design, which limits causal inference, and the reliance on self-reported outcomes, which may be subject to recall and social desirability bias.

## Background

Bangladesh has made notable progress in reducing maternal and infant mortality, yet achieving the Sustainable Development Goal (SDG) targets by 2030 remains a challenge (1, 2). Over the past two decades, Bangladesh has made remarkable progress in reducing maternal and neonatal mortality, with annual declines exceeding 4% since 2000. The maternal mortality ratio decreased from 322 per 100,000 live births in 2000 to approximately 195 during 2009–2015, while neonatal mortality fell from 42.4 per 1,000 live births in 2000 to 29.6 by 2017 (3, 4). More recently, maternal mortality has further declined to 115 per 100,000 live births, and neonatal mortality to 18 per 1,000 live births (5). However, progress in maternal and neonatal outcomes has not been matched by equitable access to antenatal care, with only approximately 44% of women achieving the recommended four or more antenatal care (ANC) visits and wide regional disparities persisting (6). Despite several programs to improve maternal and child health, gaps in healthcare-seeking and service utilization persist, with antenatal care, facility-based deliveries, and essential newborn care remaining suboptimal, especially in resource-limited settings (7). Cultural and socioeconomic barriers, along with limited awareness and access to healthcare services, contribute to these challenges, underscoring the need for innovative approaches to improve maternal and child health (MCH) outcomes (8).

Globally, religious-based interventions have demonstrated success in improving health outcomes, particularly in settings where religious beliefs significantly influence health-seeking behaviors (9–12). Religious leaders in low- and middle-income countries (LMICs) play a central role in shaping social norms, values, and health-related attitudes because of the trust and moral authority they hold within communities (13–16). Because of their role in guiding social norms, they can strongly affect how communities perceive health practices, particularly in contexts where cultural and religious values drive decision-making (17–21). This influence extends to MCH as well, where their involvement has been shown to provide a culturally sensitive and sustainable approach to improve knowledge, attitudes, and behaviors regarding antenatal care, safe delivery, and child health in several LMICs (13, 22–24). Evidence from Africa demonstrates that training and engaging religious leaders can lead to greater acceptance of maternal health services and increased use of institutional delivery and postnatal care (23–25). Similarly, in South Asia, involving religious leaders has been found to improve the acceptance of family planning, immunization, maternal health services, and efforts to reduce child marriage (26–28). This evidence indicates that religious endorsement can enhance community trust and uptake for MCH related care.

In Bangladesh, religious beliefs and institutions also play crucial roles in shaping health-related attitudes and behaviors. Religious leaders, particularly Imams (Muslim religious leaders), are highly influential figures in their communities. They engage with the public through religious teachings, advisory roles, and community gatherings, and this involvement has been shown to be beneficial in promoting public health messages, such as for non-communicable diseases (29–31). Given their widespread reach and trusted status, religious leaders also present a unique opportunity to disseminate life-saving MCH information effectively in the country. In recognition of this potential, the Islamic Foundation Bangladesh (IFB), in collaboration with the Ministry of Religious Affairs (MoRA) and UNICEF, has initiated a training programme for Imams and Islamic religion-based primary education teachers (32, 33). The initiative aims to increase the capacity of religious leaders to promote key health behaviors, including ANC utilization, facility-based deliveries, and essential newborn care, through community engagement and social mobilization, with a particular focus on poor-performing, disaster-prone, and hard-to-reach areas. However, despite the strong influence of religious leaders, there is limited population-level evidence in Bangladesh on whether structured training of religious leaders leads to measurable improvements in maternal and child health knowledge, attitudes, and practices. This study addresses this gap by providing quasi-experimental evidence on the effectiveness of a national religious leader training program in improving key MCH behaviours in underserved settings. Underserved settings in MCH refer to areas with geographic constraints and populations, including marginalized communities, that have limited access to healthcare resources due to socioeconomic disparities and weak infrastructure and transportation. They result in poor health outcomes for mothers and children (3, 6, 8, 36).

As this training program for religious leaders has plans and potential for scale-up in promoting MCH care, it is important to assess its population-level impacts. This study aims to evaluate the impact of the IFB training program by assessing community-level changes in knowledge, attitudes, practices, and behaviors (KAPB) related to key aspects of maternal and child healthcare. While some evidence has demonstrated the potential of religious leaders in enhancing health literacy for certain public health issues, there remains limited evidence on their role in influencing health-seeking behaviors in culturally sensitive areas like maternal and child health (31, 34). The integration of religious leaders in public health programs remains underexplored in Bangladesh, despite their potential to bridge healthcare gaps in hard-to-reach communities. By evaluating changes before and after the training, this study contributes to filling a critical knowledge gap and provides evidence-based recommendations for health initiatives involving religious leaders in Bangladesh, with an emphasis on promoting health equity.

## Methods

### Study design

This quasi-experimental study used repeated cross-sectional surveys conducted before and after the Islamic Foundation Bangladesh (IFB) training programme for religious leaders. We chose a repeat cross-sectional design rather than a longitudinal approach because it allows assessment of population-level changes over time across large, diverse communities, without requiring follow-up of the same individuals, which can be challenging in hard-to-reach and highly mobile populations. The training functioned as a natural intervention in the designated study areas, enabling assessment of its population-level impact on maternal and child health–related knowledge, attitudes, practices, and behaviours (KAPB).

### Intervention: IFB training program for religious leaders

The intervention was implemented by the Islamic Foundation Bangladesh (IFB) under the Ministry of Religious Affairs, with support from UNICEF, to strengthen the capacity of religious leaders in Bangladesh to promote key public health and social development messages. The program aimed to train imams and female religious-school teachers to advance community awareness on priority issues, including maternal and child health, ending child marriage, exclusive breastfeeding, early childhood care, healthy WASH practices, and gender-responsive social norms.

Training was module-based and delivered through the IFB Imam Training Academy. The curriculum incorporated interpersonal communication, community engagement, child rights, maternal and newborn health promotion, and behavior-change strategies. A training-of-trainers (TOT) approach was used, through which master trainers prepared imams and female teachers to deliver community-level messaging.

The intervention targeted religious leaders across eight districts, prioritizing poor-performing and underserved areas. IFB training is planned to cover 10,000 imams and mosque-based preprimary education teachers in 8 districts (Dhaka, Rangamati, Moulovibazar, Sirajganj, Kurigram, Jamalpur, Patuakhali and Shatkhira) through cascading to engage them in community engagement with enhanced knowledge, skills and capacity. All the mentioned districts are poor performing, disaster prone and remote districts of Bangladesh and UNICEF priority areas for intervention. Participants for training were selected from mosque-based networks in each intervention district. After the baseline survey in 2019, imams and female teachers in these communities received the training before the endline assessment period. Training duration varied by component; the core IFB Basic Training Course spanned 45 days and covered religious education alongside modules on reproductive health, family planning, gender equity, HIV/AIDS awareness, prevention of child marriage, and broader socioeconomic development topics. The religious leaders training in the selected study clusters were completed within 6 months following the baseline. The overview of the religious training intervention is displayed in Table 1.

**Table 1:** Summary of the IFB religious leader training intervention.

Component	Description
Implementing agencies	Islamic Foundation Bangladesh (IFB), Ministry of Religious Affairs; supported by UNICEF
Aim of intervention	Build capacity of religious leaders to promote health and social development behaviors, including MCH, WASH, child protection, and prevention of child marriage
Training structure	Module-based curriculum delivered through IFB Imam Training Academy; included interpersonal communication, community engagement, and behavior-change strategies
Approach	Training-of-trainers (TOT) model; master trainers trained imams and female teachers, who then disseminated messages in communities
Participant selection	Imams and female mosque-based teachers selected from mosque networks in eight priority districts
Training timeline	Training conducted after baseline (2019) and before the endline survey (2021)
Training duration	Core Basic Training Course: 45 days, covering religious studies plus public health and social development topics
Geographic coverage	Eight districts, prioritizing poor-performing, underserved, and hard-to-reach areas
Scale/targets	Planned reach: 10,000 imams; TOT completed by ∼400 imams and ∼200 female teachers; subsequent community engagement conducted by ∼2,000 imams and ∼1,600 female teachers
Key content areas	Maternal and child health including components on ANC, ENC, and facility delivery, gender equality, child marriage prevention, WASH, HIV/AIDS awareness, social protection, anti-corruption
Role in this study	Natural, policy-driven intervention implemented between baseline and endline household surveys

**Figure 1:**
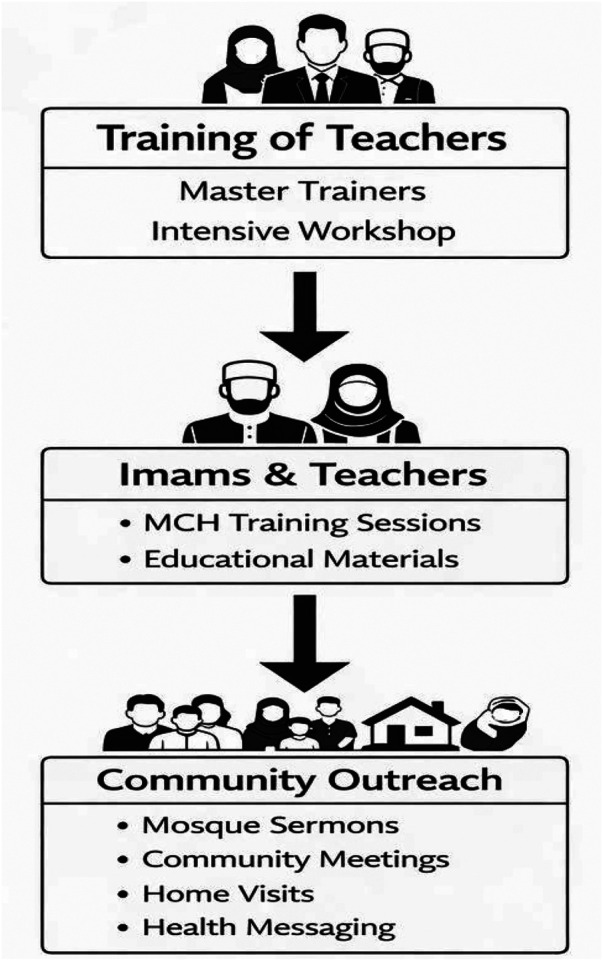
Schematic figure summarizing the training flow (TOT → imams/teachers → community).

Overall, the IFB–UNICEF program functioned as a natural policy-led intervention, equipping both male and female religious leaders with skills to disseminate structured health and social development messages within their communities (Figure 1).

### Study setting

Bangladesh is divided into eight major administrative divisions. From each division, one district was purposively chosen where the IFB had planned to conduct religious leader training during the study period. Within each district, one union council (the lowest administrative rural unit) and one municipality (the lowest administrative urban unit) were selected on the basis of their designation as the catchment area of a randomly selected religious leader trained under the IFB program. Each union council and municipality served as a study cluster. In addition to the Dhaka metropolitan city, the study included a total of 17 clusters, with two clusters from each of the eight selected districts.

### Study participants, sample size, and sampling

The study participants were selected on the basis of two criteria:

1. Direct recipients of the intervention: These were religious leaders, including imams who lead prayers at mosques and female teachers of mosque-based preprimary education programs.
2. Indirect beneficiaries: These included community members, specifically parents with children aged 0–2 years residing in the catchment area of the mosques where trained imams are expected to deliver health messages.

For direct recipients, an expected 30% increase in MCH-related KAPB following training was assumed. This assumption was based on evidence from previous studies showing similar improvements in knowledge and practices among trained community health influencers (35, 36). On the basis of a 95% significance level and 80% power, the required sample size was 789, but for robustness, 800 imams and 800 female teachers were selected at baseline and endline. For indirect beneficiaries, an expected 20% increase in MCH-related KAPB was assumed. With a 95% significance level, 80% power, and adjustment for the cluster effect, the required sample size was 1,771, and 2,000 parents (250 per district) were selected at baseline and endline. Since the community (cluster) was the measurement unit, different individuals were interviewed at baseline and endline, resulting in 3,808 participants at baseline and 3,746 at endline.

**Figure 2:**
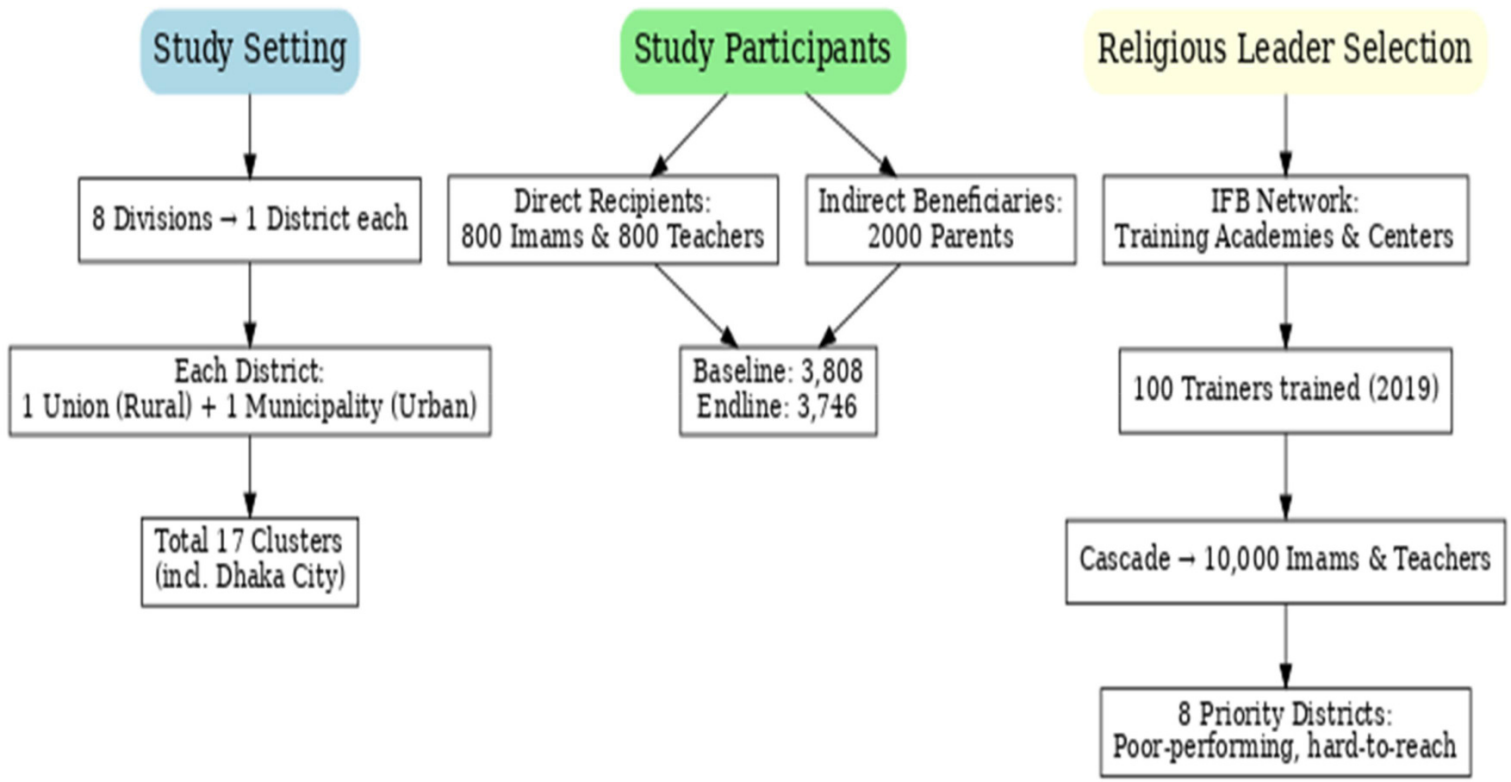
Overview of study setting, participant enrollment, and religious leader training cascade. **(a)** Change in Knowledge, Attitude, and Practice (KAP) on ANC among parents at baseline and endline. (baseline, *n* = 2,106, endline, *n* = 2,114). **(b)** Change in Knowledge, Attitude, and Practice (KAP) on facility delivery among parents at baseline and endline. (baseline, *n* = 2,106, endline, *n* = 2,114). **(c)** Change in Knowledge, Attitude, and Practice (KAP) on ENC among parents at baseline and endline. (baseline, *n* = 2,106, endline, *n* = 2,114). **(d)** Change in religious leader behaviour in disseminating maternal and child health information at baseline and endline. (baseline, *n* = 1,702, endline, *n* = 1,632).

For both the baseline and the endline, imams and female teachers were selected through simple random sampling from the list who were listed as recipients of the training under the IFB. Community people were also chosen from the catchment area through simple random sampling. Figure 2 displays the study setting, participant enrollment, and training cascade for religious leaders.

### Data collection and instrument

The baseline survey was conducted from April to June 2019, followed by a two-year IFB training program in the selected clusters. The endline survey was conducted from September to November 2021.

Three trained data collectors (one male, one female and one Imam) were selected from each district for cultural relevance and familiarity with the local context. They received extensive training on the data collection tool, which was developed on the basis of a comprehensive literature review. The structured questionnaire was designed and deployed electronically via REDCap software on tablets, with data securely stored on a protected server. The questionnaire included a sociodemographic section for both baseline and endline participants, covering age, sex, education, marital status, and residential area. Additionally, KAPB indicators related to antenatal care (ANC), childbirth practices, and essential newborn care were measured. Each interview took between 50 to 60 minutes.

### Statistical analysis

Descriptive analysis was performed to summarize the frequencies and percentages of KAPB indicators within each domain. Under each domain, individual scores were calculated for knowledge, attitudes, practices, and behavior. Table 2 illustrates the scoring procedure based on the answers to the questions posed to the participants. Multivariable logistic regression analysis was performed to compare scores in each domain from baseline to endline, adjusting for covariates (gender, age, education, and occupation). Clustering at the community level was accounted for by using cluster-adjusted logistic regression models, incorporating the design effect and intra-cluster correlation. Level of significance was ascertained at *p* < 0.05. IBM SPSS v29 was used for data analysis.

i. **Knowledge:** Participants were asked three “yes” or “no” questions related to antenatal care (ANC) and essential newborn care. Correct answers were scored as 1 and incorrect answers as 0, giving each respondent a possible total score between 0 and 3. Based on this, scores were categorized as poor (0), average (1–2), or good (3). For child delivery care, four questions were included, with possible scores ranging from 0 to 4. The categorization was defined as poor (0–1), average (2), and good (3–4).
ii. **Attitude:** Three items, each measured on a five-point Likert scale (strongly disagree = 0, disagree = 1, neutral = 2, agree = 3, strongly agree = 4), were used to assess attitudes toward ANC, delivery, and essential newborn care. For analysis, the scale was recoded: strongly disagree and disagree (0,1) were grouped as 0, while neutral, agree, and strongly agree (2,3,4) were grouped as 1. Based on this recoding, respondents’ total scores were categorized as low (0), average (1–2), or good (3).
iii. **Practice:** Correct practice was scored as 1, and no or incorrect practice as 0.
iv. **Behavior:** Imams and female teachers informed family members, relatives, and the wider community about ANC visits, delivery care, and essential newborn care through multiple channels, including Khutba, mosque discussions before or after prayers, waz-mahfi, tabliq gatherings, family and female Islamic gatherings, community meetings, schools, one-on-one conversations, and other methods. Each channel was assigned one point, resulting in a total score ranging from 0 to 10. Scores were then categorized as poor (0–2), average (3–4), or good (≥5).

**Table 2:** Scoring process based on the responses of the questions asked to the respondents.

KAPB scoring	Antenatal care	Child delivery	Essential newborn care
Knowledge	i)Heard about ANC: yes = 1, no = 0 ii)Mention the ANC services: (≥4)services = 1, (0-3) services = 0 iii)Number of ANC services should be taken during pregnancy:(≥4)-minimum four = 1,(0–3)services = 0	i)Place of conduction of delivery: At health facility (any type) = 1,home and others = 0 ii)Delivery conduction by: doctor/nurse/midwife/trained health worker = 1,others = 0 iii)Risks of home delivery: any risk = 1,no = 0 iv)Benefits of institutional delivery: any benefit = 1,no = 0	i)Mentioned essential newborn cares:(≥3)cares = 1, 0–2 cares = 0 ii)Ways to establish mother-child bonding: any contact = 1,no = 0 iii)Time to start breastfeeding:within1 hour of birth = 1, others = 0
Attitude	i)ANC is a necessity ii)ANC is necessary for both mother and child iii)Minimum 4 ANC are required	i) It is alright to deliver a child at home without assistance from skilled person. ii) It is essential to deliver a child at health facility. iii) It is essential to deliver a child by a skilled birth attendant*.*	i)It is necessary to wrap newborn with dry cloth ii)It is necessary to bathe child after 72 h of birth iii)It is necessary to breastfeed within an hour of birth
Practice	Received any ANC services: yes = 1, no = 0	Family member had their last delivery: health facility = 1, home and others = 0	Gave bathe to child: after 72 h = 1,others = 0
Behavior	Suggested/disseminated messages to family member/relatives/community/for ANC visit in any health facility: yes = 1,no = 0	Disseminated messages about the importance of an institutional delivery/delivery with skilled health personnel: yes = 1,no = 0	Disseminated messages about essential new born care: yes = 1,no = 0

## Results

### Sociodemographic characteristics of the participants

A total of 3,808 participants were surveyed at baseline and 3,746 at endline. The distribution included imams, Islamic education teachers, fathers, and mothers at both time points. The mean age of respondents decreased slightly from 34.93 ± 9.65 years at baseline to 34.41 ± 9.13 years at endline. Rural participants constituted 59.9% at baseline and 61.3% at endline. The proportion of married participants increased slightly, from 94.5% to 95.5%.

Educational attainment shifted modestly between surveys. Participants with primary education or less decreased from 49.3% to 45.1%, while those with secondary education increased from 20.4% to 23.8%. Higher secondary and graduate-and-above categories remained relatively stable. Among religious leaders, the proportion reporting prior basic imam training decreased from 24.2% to 22.7%, while those reporting other types of training increased from 29.8% to 37.9%. (Table 3)

**Table 3:** Sociodemographic characteristics of the respondents (*n* = 7,554).

Characteristics	Before intervention/Baseline *n* = 3,808	After intervention/Endline, *n* = 3,746	Statistical test	*p* value
Age				
Mean, SD	34.93 ± 9.65	34.41 ± 9.13	*t* test	.022
95%CI	34.62–35.24	34.11–34.70		
Area				
Urban	1,528 (40.1)	1,451 (38.7)	*χ* ^2^	.112
Rural	2,280 (59.9)	2,295 (61.3)		
Marital status				
Married	3,597 (94.5)	3,577 (95.5)	*χ* ^2^	.023
Others	211 (5.5)	169 (4.5)		
Educational level				
Up to Primary school	1,887 (49.3)	1,688 (45.1)	*χ* ^2^	.000
Secondary school	777 (20.4)	890 (23.8)		
Higher Secondary school	441 (11.6)	444 (11.9)		
Graduate and above	712 (18.7)	724 (19.3)		
Training for religious leader (*n* = 3,334)				
Basic imam training (IFB)	412 (24.2)	371 (22.7)	*χ* ^2^	.000
Other Training	508 (29.8)	618 (37.9)		
No training	782 (45.9)	643 (39.4)		
Total	1,702(100)	1,632(100)		

Variable description: Other training- English language training, basic computer skill training.

### Changes in maternal and child health-related knowledge, attitudes, practices, and behavior outcomes

**Figure 3:**
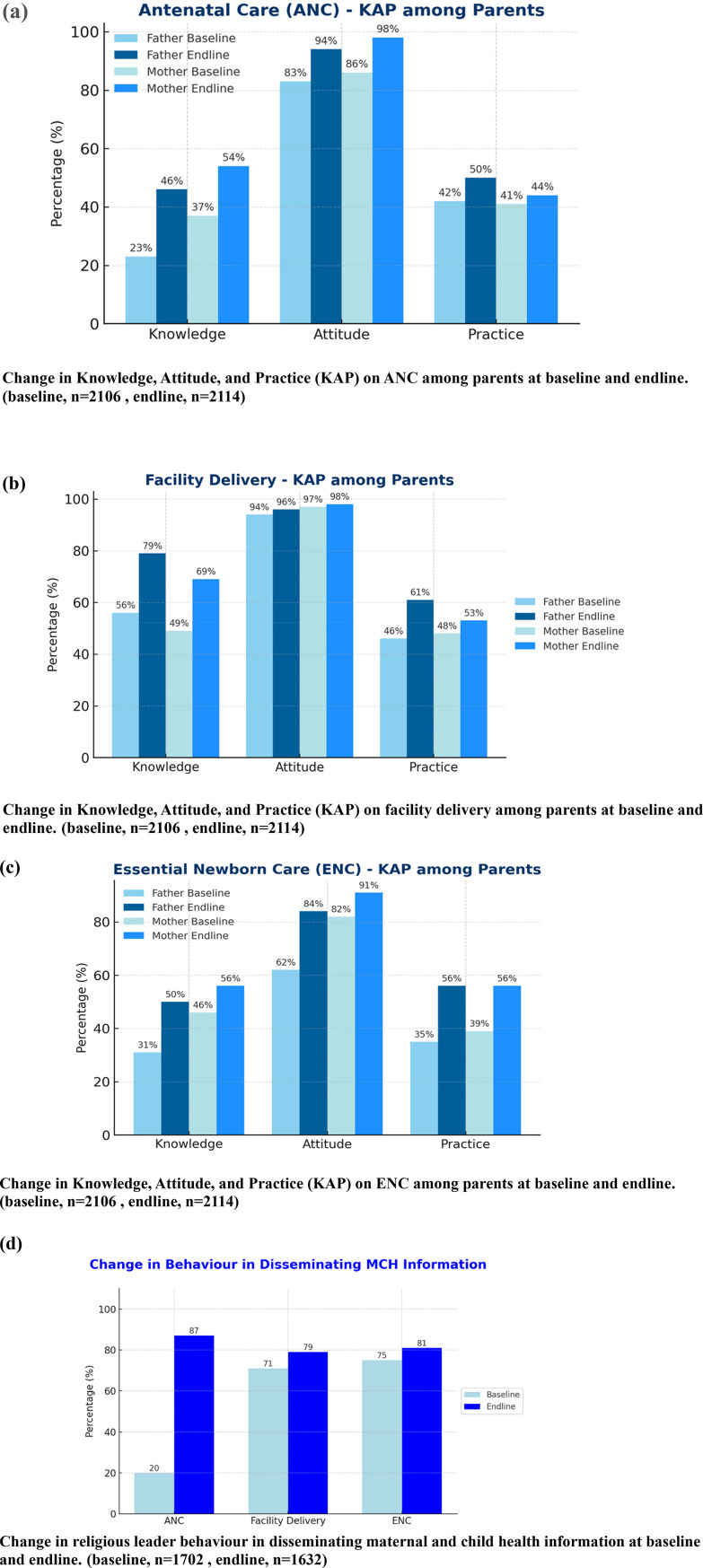
Changes in maternal and child health-related knowledge, attitudes, practice, and behaviour outcomes following the intervention. **(a)** Change in Knowledge, Attitude, and Practice (KAP) on ANC among parents at baseline and endline. (baseline, *n* = 2106, endline, *n* = 2114). **(b)** Change in Knowledge, Attitude, and Practice (KAP) on facility delivery among parents at baseline and endline. (baseline, *n* = 2106, endline, *n* = 2114). **(c)** Change in Knowledge, Attitude, and Practice (KAP) on ENC among parents at baseline and endline. (baseline, *n* = 2106, endline, *n* = 2114). **(d)** Change in religious leader behaviour in disseminating maternal and child health information at baseline and endline. (baseline, *n* = 1702, endline, *n* = 1632).

Figure 3 illustrates baseline and endline distributions of maternal and child health–related knowledge, attitude, practice, and behavioural dissemination scores among survey respondents. Across all components, the endline bars show a higher proportion of participants classified in the “good” category than at baseline. For antenatal care, the proportion of respondents with good knowledge increased from baseline to endline, consistent with the rise observed in practice scores, where endline respondents more frequently reported receipt of ANC services. For delivery care, the figure shows a noticeable increase in the proportion achieving good knowledge at endline, accompanied by higher practice scores relative to baseline. Essential newborn care similarly shows an upward shift, with more respondents meeting the criteria for good knowledge and good practice at endline, including behaviours such as early breastfeeding and delayed bathing. Dissemination behaviour also increased across domains, as reflected in higher endline bars for message-sharing activities; for example, imams’ dissemination of ANC information increased from 39% to 88%, and dissemination of newborn-care messages increased from 79% to 82%. Taken together, the figure displays a general upward shift in the distribution of KAPB scores, with endline values consistently exceeding corresponding baseline values across all measured components.

**Figure 4:**
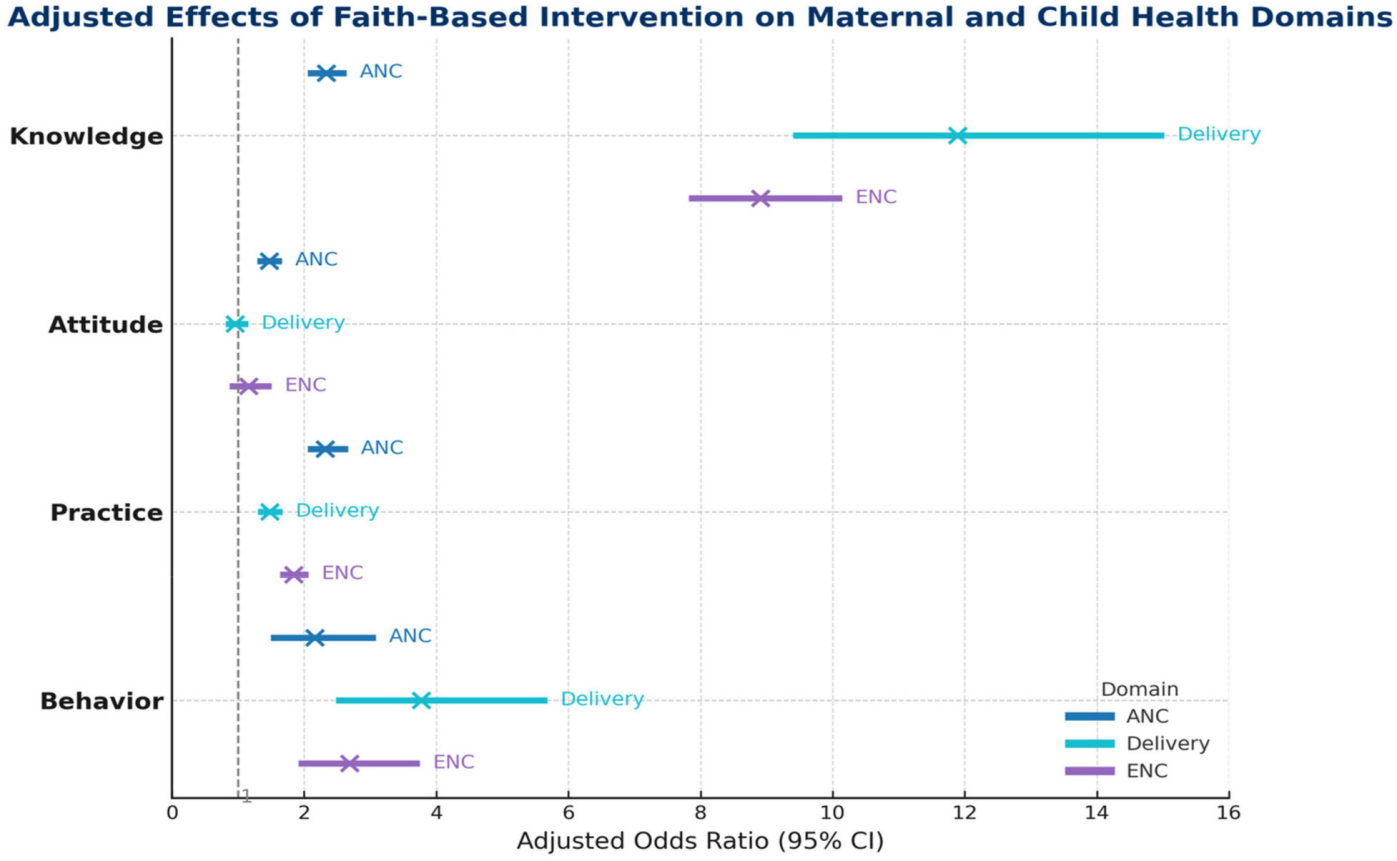
Impact of religious leader training on ANC, facility-delivery, and ENC related knowledge, attitude, practice, and behaviour in the communities. (baseline, *n* = 3,808, endline, *n* = 3,746.

Figure 4 presents adjusted odds ratios comparing endline with baseline across all maternal and child health indicators after adjustment for age, sex, education, and occupation. The figure includes outcome domains defined by the study's scoring system. For antenatal care (ANC), knowledge reflects the respondent's ability to correctly identify key recommendations such as the need for at least four ANC contacts; attitude reflects agreement that ANC is necessary; and practice represents reporting receipt of any ANC service. At endline, adjusted odds ratios were higher for all ANC components, including knowledge (aOR 2.3, 95% CI 2.1–2.6), positive attitudes (aOR 1.5, 95% CI 1.4–1.6), and practice (aOR 2.3, 95% CI 2.1–2.6). For facility delivery, knowledge refers to identifying risks of home delivery and benefits of institutional delivery, attitude reflects preference for facility-based childbirth, and practice represents reporting a facility delivery at the last birth. The figure shows a large increase in delivery-related knowledge (aOR 11.9, 95% CI 9.5–14.9) and a moderate increase in practice (aOR 1.5, 95% CI 1.4–1.6), while attitudes remained unchanged (aOR ∼1). For essential newborn care (ENC), knowledge includes understanding correct practices such as early initiation of breastfeeding, delayed bathing for at least 72 h, and thermal care; attitude reflects agreement with these recommended practices; and practice indicates engagement in these behaviours. Adjusted odds were higher for ENC knowledge (aOR 8.9, 95% CI 7.8–10.1) and practice (aOR 1.8, 95% CI 1.7–2.0), whereas attitudes remained stable. Behavioural dissemination indicators in the figure show higher adjusted odds at endline for sharing ANC messages (aOR 2.2), delivery-care messages (aOR 3.7), and newborn-care messages (aOR 2.7). Overall, the figure depicts higher adjusted odds for most measured components.

## Discussion

This study provides evidence from, to our knowledge, the first large-scale population-level evaluation of a religious leader training programme on maternal and child health (MCH)–related knowledge, attitudes, practices, and behaviours in Bangladesh. Using repeated cross-sectional surveys before and after implementation of the Islamic Foundation Bangladesh (IFB) training, we observed substantial gains in antenatal care (ANC)–related knowledge, attitudes, and service use; marked improvements in knowledge of delivery risks and essential newborn care (ENC) practices; modest but significant improvements in facility-based delivery; and large increases in reported health message dissemination by imams and female religious-school teachers. These effects were observed in socio-demographically comparable baseline and endline populations, suggesting that the training, operating as a natural policy-led intervention, was associated with meaningful shifts in community-level MCH-related KAPB in underserved districts.

Across domains, knowledge gains were substantial and statistically significant. For ANC, endline participants had more than twice the odds of correctly identifying key recommendations, including the need for at least four ANC contacts, and showed higher composite knowledge scores. For delivery care, knowledge of home-delivery risks and benefits of institutional delivery increased nearly twelve-fold, and ENC knowledge, including early breastfeeding initiation, delayed bathing, and thermal care, also rose markedly. Communities in Bangladesh are highly religiously inclined and regular attendance at mosque-based sermons and khutbas is common, repeated exposure to health messages through these trusted platforms may have contributed to the substantial gains in knowledge. Embedding health content within religious teaching may have enhanced salience and recall, particularly in rural and hard-to-reach settings where imams and religious teachers are primary sources of trusted information (13, 14, 19, 29).

Comparable patterns have been reported in other faith-based or religious leader–centred interventions. Reviews from Africa indicate that faith-based organisations and religious actors can improve maternal and newborn health knowledge and awareness when they are equipped with tailored curricula and communication tools (23, 25). A cluster-randomised trial in northern Ghana that mobilised faith-based and lay leaders around ANC showed improvements in knowledge of danger signs and preparedness for birth and newborn care (24). Similarly, work from Tanzania and other settings has shown that training religious leaders can enhance knowledge and counselling around family planning and related health issues (20, 22). In South Asia, initiatives engaging Muslim religious scholars and faith leaders have reported improvements in community knowledge and discourse around maternal and newborn health and child marriage (26–28, 37). In Bangladesh, prior programmes using mosques to disseminate messages on second-hand smoke and diabetes prevention also documented improved community awareness and risk perception, underscoring the potential of religious platforms for health literacy (30, 31). Our findings extend this evidence by demonstrating population-level gains in MCH-specific knowledge following a nationally implemented training in an LMIC context.

In contrast to knowledge, attitudinal change was more modest and domain-specific. ANC-related attitudes, such as agreement that ANC is necessary and beneficial for both mother and child, improved significantly, whereas attitudes towards facility delivery and ENC showed little change. This pattern is consistent with the understanding that knowledge may respond more quickly to information provision, while attitudes and norms around childbirth and newborn care are shaped by deeper sociocultural expectations, gender roles, and intergenerational practices (38–40). Previous work from South Asia has highlighted that religious affiliation and normative beliefs can both support and hinder MCH-related attitudes and care seeking, depending on how religious messages interact with local gender and family dynamics (21, 34). Studies that have documented larger shifts in attitudes often combined religious leader engagement with intensive community dialogue, women's groups, or broader social mobilisation strategies (24, 41, 42). The relatively limited change in attitudes around delivery and ENC in this study suggests that single-channel interventions, even when trusted, may need to be complemented by longer-term, multi-layered strategies to transform deeply embedded norms.

Practice outcomes also improved, particularly for ANC use and ENC behaviours. Participants at endline were more than twice as likely to report receiving any ANC service compared with baseline, and ENC practices such as early breastfeeding initiation and delaying the first bath by at least 72 h were significantly more common. Facility-based delivery increased to a lesser but still meaningful extent, with an adjusted odds ratio of 1.5 for reporting a facility delivery at the last birth. These findings reflect partial translation of knowledge and religiously endorsed messages into concrete care-seeking and caregiving behaviours along the MCH continuum.

The pattern of stronger gains for ANC and ENC practices than for facility delivery is understandable in the Bangladeshi context. ANC visits and early breastfeeding can often be adopted with relatively fewer financial or logistical barriers, particularly when encouraged by respected religious leaders and aligned with existing health system provision. By contrast, facility delivery is more constrained by distance, cost, transport, and perceived quality of care, all of which have been documented as barriers in Bangladesh and other LMICs (7, 8, 16, 38). Therefore, while religious endorsement may increase acceptability and intention, structural barriers may limit the absolute magnitude of change in delivery practices.

International evidence supports this differentiated pattern. Studies of faith-based and religious leader–led interventions have reported consistent improvements in ANC uptake and selected newborn practices, but more variable effects on facility delivery and skilled birth attendance, especially where health system access is limited (23, 25). For example, a trial in Ethiopia that engaged religious leaders alongside community health workers observed increases in ANC use and facility-based delivery, but the latter gains were more modest and highly context-dependent (41–43). Similarly, evidence from northern Ghana and other sub-Saharan African settings shows improvements in preparedness and some behaviours, but persistent challenges in overcoming structural barriers to institutional childbirth (24, 43). Our findings align with this literature: religious leaders can help close “acceptability” gaps and support earlier and more appropriate care, but to fully shift delivery patterns, health system accessibility and quality must also improve (25, 38).

The improvements in ENC practices observed here, particularly early initiation of breastfeeding and delayed bathing, are noteworthy. Such behaviours are often tightly linked to social and religious norms about purity, warmth, and maternal roles. When religious leaders explicitly endorse evidence-based newborn care as consistent with religious values and child protection, families may feel more confident in departing from harmful traditional practices. This mechanism is reflected in previous work that highlights the role of faith leaders in reframing health behaviours as moral and religiously appropriate actions, thereby facilitating adoption (17, 23, 26, 37). Our data suggest that this mechanism may be particularly relevant for behaviours performed at home, where religious and cultural guidance is central to decision-making.

Behavioural dissemination by religious leaders themselves showed some of the largest relative changes. Between baseline and endline, the proportion of imams reporting that they disseminated ANC messages increased from 39% to 88%, with similar improvements for delivery and ENC messages. Female religious-school teachers also reported higher engagement in message dissemination. In adjusted analyses, the odds of disseminating messages on ANC, facility delivery, and ENC at endline were approximately two- to four-fold higher than at baseline. These findings indicate that the training not only increased knowledge but also translated into more frequent and diversified communication by religious leaders across multiple platforms, including sermons, mosque announcements, community meetings, and individual counselling.

This pattern is consistent with evidence that religious leaders can function as highly active “communication hubs” when they are provided with clear, context-appropriate content and encouraged by formal structures such as ministries of religious affairs and national faith-based organisations (14, 15, 37, 44, 45). Studies from Ghana, Tanzania, and Ethiopia show that when religious leaders are integrated into structured outreach or home-visiting models, they can significantly expand the reach of health messages and complement routine health worker activities (22, 24, 41, 42). In West Africa and northern Nigeria, involving imams in communication strategies has also been critical in rebuilding trust after vaccine boycotts and improving immunisation uptake (25, 46). During the COVID−19 pandemic, religious leaders in several LMICs, including Bangladesh, mobilised to support public health guidance, illustrating their agility and centrality in crisis communication (15, 33, 37). Our findings add quantitative, population-level evidence from Bangladesh to this literature, demonstrating that a national, government-led training programme can substantially change the communication behaviour of religious leaders in routine, non-emergency MCH domains. The observed increases in message dissemination also resonate with broader work on strategic health communication in low-resource settings, which emphasises the need to combine mass media with trusted interpersonal channels to achieve sustained behaviour change (43, 45, 47). In this study, imams and female religious teachers effectively became locally embedded “behaviour change agents,” amplifying formal programme content and potentially reinforcing health messages delivered through other channels.

Overall, this study suggests that a structured, nationally implemented training of religious leaders in Bangladesh was associated with meaningful improvements in maternal and newborn health–related knowledge, practices, and communication at the community level, with more modest and domain-specific shifts in attitudes. The findings reinforce the potential of religious leaders as strategic partners in MCH programming, particularly in underserved settings where religious institutions are deeply embedded and trusted. Integrating such engagement into national health communication and community strategies, alongside efforts to address structural barriers to care, may help accelerate progress toward maternal and newborn health targets and contribute to more equitable outcomes across hard-to-reach communities (1, 2, 23, 25, 37, 45).

### Strengths and limitation of the study

This study has several methodological and contextual strengths that enhance the reliability and policy relevance of its findings. First, it utilized a repeated cross-sectional design with large, representative samples drawn from multiple administrative divisions of Bangladesh, thereby increasing the generalizability of the results across diverse geographical and sociocultural settings. The sampling strategy, which included both direct recipients of the intervention (religious leaders) and indirect beneficiaries (community members), allowed for a comprehensive assessment of the intervention's population-level effects. Second, the use of a structured and pretested questionnaire based on established KAPB frameworks ensured consistent measurement of maternal and child health-related indicators across time points. Data collection through electronic platforms minimized data entry errors and improved the overall quality and completeness of the dataset. Importantly, the regression models were adjusted for key sociodemographic variables, reducing potential confounding factors and increasing the validity of the observed associations.

Despite its strengths, the study has several limitations. The repeated cross-sectional design limits causal inference, as different individuals were surveyed at baseline and endline. The outcomes were assessed by comparing individuals who had exposure to the intervention with those who did not. As a result, the estimated effects reflect the intervention's effectiveness at the community level, rather than its efficacy specifically among the individuals who directly participated. Longitudinal data would better capture individual-level changes. Self-reported data may be subject to social desirability bias, especially given the involvement of religious leaders. The study did not measure clinical outcomes like maternal or neonatal morbidity, limiting conclusions about health impact. Additionally, unmeasured confounders such as other community interventions or health system changes may have influenced the results. Finally, our cross-sectional pre- and post-design could not capture long-term sustainability of the observed changes.

While the findings from this study are promising, several areas need further exploration. Future research should consider longitudinal designs to assess whether the observed improvements are sustained over time and whether they translate into improved maternal and neonatal health outcomes. Further research is needed to assess the cost-effectiveness of religious-based interventions relative to other community-based behavioral change approaches, which could guide resource allocation in low- and middle-income settings. Moreover, an analysis of gender-specific impacts and the role of female religious teachers in reaching women within the household and community contexts could help refine future program designs. There is also scope for evaluating the integration of digital communication tools to augment the reach and frequency of health messages delivered by religious leaders. Religious leaders are likely to sustain health messaging due to their trusted community roles, though periodic support may be needed. Potential social desirability and recall biases are acknowledged, and all data were anonymized and securely handled to ensure participant confidentiality.

## Conclusion

This study provides strong empirical support for the use of religious leader training as a viable strategy to increase maternal and child health-related knowledge and practices at the community level in Bangladesh. Training religious leaders as community change agents can enhance culturally responsive engagement and accelerate progress toward SDG 3 maternal and neonatal health targets. Integrating C4D within existing MNCH programs, alongside strengthened collaboration between religious leaders and frontline health workers, may improve continuity of care and timely use of ANC, delivery, and postnatal services. Emphasizing context-specific communication and strong monitoring frameworks will be critical to sustaining impact and informing scale-up. Although additional efforts are required to institutionalize and expand the intervention, the findings demonstrate a promising, culturally aligned model for sustainable health promotion.

## Data Availability

The raw data supporting the conclusions of this article will be made available by the authors, without undue reservation.
