# Supplementary material for: Impacts of religious leader training on maternal and child health in underserved communities of Bangladesh
Source: Front Reprod Health. 2026 Apr 9;8:1764081. doi: 10.3389/frph.2026.1764081 (PMC13102671; doi:10.3389/frph.2026.1764081)
Supplement: Supplementary file 1 [file Table1.docx]

Table: Changes in knowledge, attitudes, practices and behaviors in maternal and child-healthcare domains following the intervention.

|  | **AOR (95% CI) adjusted** | ***p* Value** |
| --- | --- | --- |
| **ANC** | | |
| Knowledge | 2.33(2.10-2.59) | .000 |
| Attitude | 1.47(1.34-1.61) | .000 |
| Practice | 2.32(2.10-2.61) | .000 |
| Behavior | 2.16(1.54-3.03) | .000 |
| **Delivery care** | | |
| Knowledge | 11.89(9.45-14.96) | .000 |
| Attitude | .95(.86-1.10) | .367 |
| Practice | 1.48(1.35-1.62) | .000 |
| Behavior | 3.77(2.53-5.63) | .000 |
| **Essential Newborn care** | | |
| Knowledge | 8.91(7.87-10.09) | .000 |
| Attitude | 1.16(.92-1.45) | .204 |
| Practice | 1.84(1.68-2.01) | .000 |
| Behavior | 2.69(1.96-3.70) | .000 |

[Adjusted for sex, age, education, and occupation]
